# The complete chloroplast genome of *Piper sarmentosum* Roxburgh, 1820 (Piperaceae)

**DOI:** 10.1080/23802359.2022.2074805

**Published:** 2022-05-12

**Authors:** Xiaoshan Geng, Yulin Zhu, Zhenxin Ren, Rong Chen, Qin Liu

**Affiliations:** Guangxi Key Laboratory of Agricultural Resources Chemistry and Biotechnology, Yulin Normal University, Yulin, China

**Keywords:** Chloroplast genome, phylogenetic analysis, *Piper sarmentosum*

## Abstract

*Piper sarmentosum* Roxb. (Piperaceae) is a traditional medicinal herb native to Southeast Asia. The complete genome of *P. sarmentosum* was sequenced and characterized in this study with the aim of providing genomic resources for the evolution and molecular breeding of *P. sarmentosum*. It has a typical quadripartite structure, with a large single-copy (LSC) region of 88,979 bp, a small single-copy (SSC) region of 18,274 bp, and two copies of 27,068 bp inverted-repeat regions (IRa and IRb). A total of 130 genes were annotated, comprising 85 protein-coding genes (PCGs), 8 ribosomal RNA (rRNA) genes, and 37 transfer RNA (tRNA) genes. The phylogenetic tree showed that *P. sarmentosum* in the current study is closely related to *Piper longum*.

*Piper sarmentosum* Roxb. (Piperaceae) is a traditional medicinal herb found mainly in Southeast Asia (Mathew et al. 2004). Due to its antioxidant and anti-inflammatory properties, it is beneficial for various diseases, including fever, toothache, dysentery, and traumatic injury (Sun et al. 2020). Its medicinal value is complemented by its edible value, as its leaves are frequently consumed as a tasty vegetable. Previous studies have demonstrated that the extracts of *Piper sarmentosum* are rich in alkaloids, essential oils, flavonoids, lignans, and steroids. The majority of current research on this plant has focused on compound isolation and identification, as well as pharmacology, but little is known about its molecular genetics, limiting its conservation and utilization. This study aims to obtain and characterize the complete chloroplast genome of *P. sarmentosum* and provide valuable genomic information for its phylogeny and molecular breeding.

The fresh leaves of *P. sarmentosum* were collected from the Yulin Normal University Horticultural Germplasm Center in Yulin, Guangxi, China (110.185°E, 22.669°N). The voucher specimen (JL-YNU-001) was deposited in the Herbarium of Yulin Normal University (https://syy.ylu.cn/index.html, Yulin Zhu, gxzyl@163.com). A modified CTAB approach (Porebski et al. 1997) was used to extract the total genomic DNA of *P. sarmentosum*. Library construction and sequencing were conducted by Biozeron (Biozeron, Shanghai, China) using the Illumina HiSeq 4000 sequencing platform (Illumina, San Diego, CA) with 150 bp paired-end (PE) sequencing. The adapters and low-quality sequences of the obtained raw reads were trimmed and filtered by the Trimmomatic software (Bolger et al. 2014) with the options of ‘LEADING:2, TRAINLING:3, SLIDINGWINDOW:4:15, MINLEN:100.’ The *de novo* assembly and annotation of the *P. sarmentosum* chloroplast genome were achieved by the GetOrganelle toolkit (Jin et al. 2020) and CPGAVAS2 (Shi et al. 2019) with default parameters, respectively. The final chloroplast genome was submitted to GenBank with accession no. MZ958833.

The chloroplast genome, with a total length of 161,389 bp, has a typical quadripartite structure, containing a large single-copy (LSC) region of 88,979 bp, a small single-copy (SSC) region of 18,274 bp, and two copies of inverted-repeat regions (IRa and IRb) of 27,068 bp each. A total of 130 genes were annotated, comprising 85 protein-coding genes (PCGs), 8 ribosomal RNA (rRNA) genes, and 37 transfer RNA (tRNA) genes. 18 of these genes have double copies, and a total of 21 genes contain introns, including 8 tRNA genes and 13 PCGs, of which *ycf3* and *clpP* contain two introns each, and the others contain only one intron.

To confirm the phylogenetic position of *P. sarmentosum*, complete chloroplast genome sequences from the Piperaceae family and several closely related families from the order Piperales were used to reconstruct a phylogenetic tree. Multiple sequence alignments based on the single-copy orthologue genes from all chloroplast genomes were performed by MAFFT v7 (Katoh and Standley 2013) with default parameters, and the maximum-likelihood tree was built using IQ-TREE v1.6.10 (Nguyen et al. 2015) with the best-fit model TVM + F + G4 and 1000 bootstrap replicates (Figure 1). Two species from the order Canellales, *Drimys granadensis* and *Tasmannia lanceolata*, were used as the outgroups. The phylogenetic tree shows that all Piperaceae species form a group, and *P. sarmentosum* in the current study is closely related to *Piper longum*. The complete chloroplast genome of *P. sarmentosum* could provide a genomic resource for further genetic and evolutionary studies on Piperales species.

**Figure 1.**
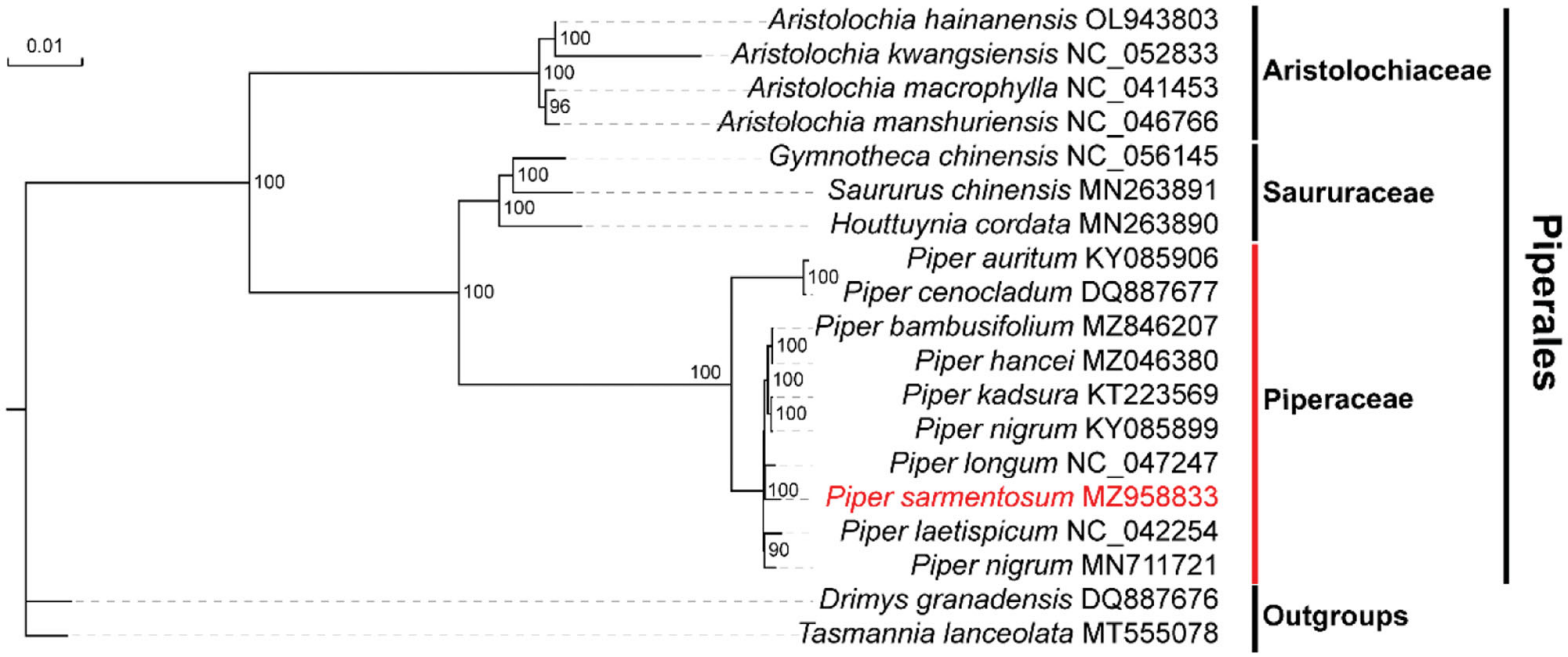
Maximum-likelihood phylogenetic tree based on 17 chloroplast genomes of Piperales and two outgroup species. Numbers at the nodes are bootstrap support values based on 1000 replicates. The species *P. sarmentosum* is highlighted in red.

## Data Availability

The genome sequence data that support the findings of this study are openly available in GenBank of NCBI at https://www.ncbi.nlm.nih.gov under the accession no. MZ958833. The associated BioProject, SRA, and Bio-Sample numbers are PRJNA818010, SRR18391781, and SAMN26814157, respectively.
